# Change in diet in the period from adolescence to early adulthood: a systematic scoping review of longitudinal studies

**DOI:** 10.1186/s12966-017-0518-7

**Published:** 2017-05-04

**Authors:** Eleanor M. Winpenny, Tarra L. Penney, Kirsten Corder, Martin White, Esther M. F. van Sluijs

**Affiliations:** MRC Epidemiology Unit & Centre for Diet and Activity Research (CEDAR), University of Cambridge School of Clinical Medicine, Institute of Metabolic Science, Cambridge Biomedical Campus, Box 285, Cambridge, CB2 0QQ UK

**Keywords:** Diet, Adolescent, Young adult, Longitudinal, Trajectory

## Abstract

**Background:**

Late adolescence to early adulthood is a period of lifestyle change and personal development which may influence dietary behaviour. Understanding dietary trajectories across this age range may help in targeting interventions appropriately. This scoping review aimed to assess how longitudinal change in diet is conceptualised and measured between the ages of 13 to 30.

**Methods:**

We searched Medline, SCOPUS, Embase, PsycInfo (EBSCO), ASSIA, Sportdiscus, and Web of Science Core Collection (January 2016) using search terms combining diet outcomes, longitudinal methods and indicators of adolescent or young adult age. Titles and abstracts were screened and data extracted following published guidelines for scoping reviews. Data were analysed to summarize key data on each study and map availability of longitudinal data on macronutrients and food groups by age of study participants.

**Results:**

We identified 98 papers reporting on 40 studies. Longitudinal dietary data were available on intake of energy, key macronutrients and several food groups, but this data had significant gaps and limitations. Most studies provided only two or three waves of data within the age range of interest and few studies reported data collected beyond the early twenties. A range of dietary assessment methods were used, with greater use of less comprehensive dietary assessment methods among studies reporting food group intakes.

**Conclusion:**

Despite limited availability of longitudinal data to aid understanding of dietary trajectories across this age range, this scoping review identified areas with scope for further evidence synthesis. We identified a paucity of longitudinal data continuing into the mid and late twenties, variability in (quality of) dietary assessment methods, and a large variety of macronutrients and food groups studied. Advances in dietary assessment methodologies as well as increased use of social media may facilitate new data collection to further understanding of changing diet across this life stage.

**Electronic supplementary material:**

The online version of this article (doi:10.1186/s12966-017-0518-7) contains supplementary material, which is available to authorized users.

## Background

During adolescence and early adulthood, poor diet contributes towards immediate health risks, such as weight gain, inadequate bone mineralization and poor academic performance [1]. However, in addition, poor diets developed in childhood frequently persist into adulthood [2], influencing risk of chronic disease in later life including diabetes, cardio-vascular disease and certain cancers [3–5]. The period of late adolescence to early adulthood has been suggested as an important but overlooked age for establishment of long-term health behaviour patterns [6].

Adolescence is a period of rapid physiological and psychological development [7], while early adulthood has been described as ‘demographically dense’, characterised by transitions between different social roles [8]. In addition to further development of self-identity during this period [9], individuals experience major transitions during the adolescent to early adulthood period. These include changes in social environment and social influences, moving from family dependence towards stronger peer networks and development of intimate partner relationships. There may be changes in the home environment, with individuals often moving out of the family home to live independently, changes in school/work environment from secondary to further education and into employment and changes in financial circumstances, with individuals often achieving financial independence during this period. Many of these factors show associations with diet and eating behaviours in this age range [10–12].

Despite tracking of diet between childhood and adulthood [2], there is evidence to support the “habit discontinuity hypothesis” which states that behaviour change is more likely to be successful when a context change disrupts individuals’ pre-existing habits [13, 14]. Adolescent to adult transitions may thus present opportunities for interventions to improve diet. In order to develop and target public health interventions most appropriately, it is important to understand underlying changes in diet throughout this period, the establishment of long-term dietary patterns, and factors that impact on changes in diet.

While a number of studies have reported on longitudinal changes in diet over the period from adolescence to early adulthood, the evidence is diverse in terms of the dietary measures of interest, methods of dietary assessment, age range of interest and frequency of follow-up [15–17]. The heterogeneous nature of this evidence can make systematic review and meta-analysis difficult, nevertheless a comprehensive overview of the literature is needed to provide an understanding of the data available and current knowledge on change in diet across this age range. The purpose of this work was to support this by conducting a systematic scoping review and synthesis to identify the range of available evidence and address the question: How is longitudinal change in diet conceptualised and measured in the period from adolescence to early adulthood (age 13–30)? Specifically, we aimed to: (1) map the cohorts providing data on longitudinal diet, by country, size, data collection period and method of dietary assessment used; (2) assess the evidence available across macronutrients and food groups; and (3) analyse the number of repeated measurements and age ranges covered by data on each macronutrient and food group. From this data synthesis we make recommendations for further systematic review and data collection to address questions concerning change in diet in this age range.

## Methods

We carried out a scoping review following published guidelines [18–21], as shown in Table 1.

**Table 1:** Scoping review definition and methodology

“A scoping review or scoping study is a form of knowledge synthesis that addresses an exploratory research question aimed at mapping key concepts, types of evidence, and gaps in research related to a defined area or field by systematically searching, selecting, and synthesizing existing knowledge.” [21]	
Stages of the scoping review methodology: [18]	
1. Identifying the research question	
2. Identifying relevant studies	
3. Study selection	
4. Charting the data	
5. Collating, summarizing and reporting the results	

We registered the review on PROSPERO (ref: CRD42015030126).

We looked for prospective longitudinal data on dietary intake reported between mean ages of 13 and 30 years. While a consensus has not been reached on a definition of early adulthood, Rindfuss [8] suggested the 30th birthday as the end of the young adult years. Adolescence is defined by the World Health Organization (WHO) as ages 10 to 19 years [22], however we begin our review at age 13 years, since we are primarily interested in transitions occurring from mid-adolescence to early adulthood. This period includes that of ‘emerging adulthood’ which has been conceptualised as covering the period from age 18 to 25 years and postulated as a transitionary period between adolescence and full adulthood [23].

We searched Medline, SCOPUS, Embase, PsycInfo (EBSCO), ASSIA, Sportdiscus, and Web of Science Core Collection in January 2016 using search terms that combined diet outcomes, longitudinal methods and indicators of adolescent or young adult age range. Since this review was conducted in parallel with a partner review looking at change in physical activity (PROSPERO ref.: CRD42015030114), the search terms also included physical activity outcomes. The full search strategy as applied to Medline is shown in Additional file 1: Table S1. We also conducted additional hand searches; checking the reference lists of all included papers, and citation searches of key included papers which addressed the same research question as this review.

Three researchers (EW, KC, EvS) screened titles and abstracts of identified records against a set of inclusion and exclusion criteria (Table 2). Firstly, the researchers independently screened the same 500 records and compared their results in order to ensure consistency in deciding on study eligibility. This process was repeated 3 times until acceptable consistency was reached (93% agreement between all three reviewers). The remaining titles and abstracts were then screened by one of the three researchers. Full texts of potentially eligible studies were retrieved, separated by behaviour of interest, after which the diet-related full text papers were assessed against the inclusion and exclusion criteria by two researchers (EW and TP) independently. There was 93% initial agreement on inclusion/exclusion and discrepancies were resolved by discussion. Any remaining disagreements or uncertainties between the reviewers were resolved by discussion within the wider research team.

**Table 2:** Inclusion and exclusion criteria for the review

	Inclusion criteria	Exclusion criteria
Setting	Any country Studies where specified data (see below) was collected during or after 1980	Studies reporting on data collected prior to 1980
Participants	Those aged between 13 and 30 years, inclusive	Those aged below 13 years of age or above 30 years of age Participant groups selected based on a pre-existing health condition (including obesity, eating disorders, malnutrition) or pregnancy Studies where participants are only competitive athletes
Outcomes	A measure of diet, including: - Energy intake - Intake of macronutrients or micronutrients - Intake of particular food groups	Studies including no dietary outcomes Studies reporting tracking of dietary behaviours only with no data on absolute change in behaviour Studies reporting solely on alcohol intake Studies reporting on eating disorders or weight reduction behaviour Studies reporting on diet quality indices or dietary patterns only
Study type	Longitudinal prospective quantitative studies, with data reported including on specified outcomes at at least 2 time points (minimum 1 year apart) where mean age of the cohort is between ages 13 and 30 inclusive	Cross-sectional studies Intervention studies/trial analyses Case-control studies Retrospective studies Qualitative studies Reviews
Publication type	Journal article	Conference abstract, study protocol, report, dissertation, book and professional journal
Language	English	All other languages

Data were extracted from each included study on the diet outcomes reported, together with details of the ages at which data were available, further details of the study population and context (the size of each cohort, country and period of data collection, the variation in age of the cohort at baseline), and the dietary assessment method used. Data extraction was undertaken by one researcher (EW) and checked by a second (TP). No formal quality assessment was undertaken following standard practice for scoping reviews [18].

Data were summarized to record the number of papers and studies retrieved by country and record key details extracted from each study, including the number of data points collected between mean ages of 13 and 30 and the mean age of the cohort at each data collection. We then focussed on mapping of data by macronutrients and food groups and, for each macronutrient or food group where 5 or more studies had reported data, graphed the availability of data from each study by mean age at each data collection point. We grouped data on foods and food groups under seven food groups. However, we note that the data collected by different studies is not always easily comparable. For example, within the category ‘dairy’ this includes both studies that report data on all dairy consumption, as well as studies reporting on consumption of specific dairy products such as milk or cheese.

## Results

**Fig. 1:**
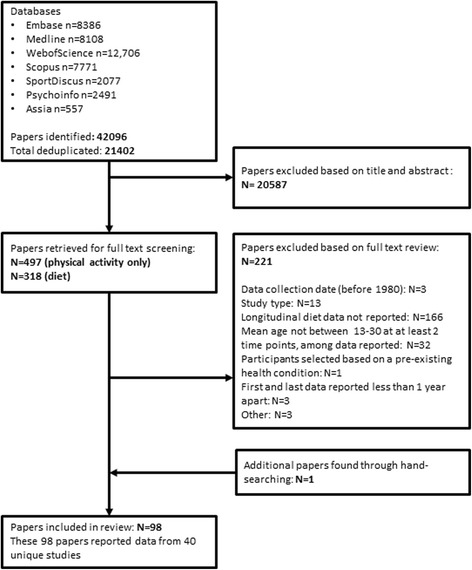
PRISMA flow diagram

Searches identified a total of 21,402 records following the removal of duplicates. After initial screening of titles and abstracts, we considered 318 references for full text review for the current scoping review. One paper was added from additional citation searches of included articles (Fig. 1). In total, 98 papers were included which reported longitudinal data on dietary intake between the ages of 13 and 30.

### Populations covered by study cohorts

The 98 papers reported data from 40 unique studies. Table 3 presents an overview of the papers and studies by the cohort’s country of origin. Studies were found reporting on data from 15 countries, with the largest number of studies (*n* = 15) originating from the US. A large number of papers were found from the Netherlands (*n* = 16), but these reported on data from only 2 studies. Similarly, 13 papers from Australia reported on 5 studies and 10 papers from Norway reported on 3 studies. From each of the remaining 11 countries we found only up to 4 papers. For each country we have listed the named studies in Table 3.

**Table 3:** Number of papers and studies retrieved by country

Country	Papers	Studies	Named studies
Australia	13	5	Adelaide Nutrition Study (ANS)
			Raine Study (The Western Australian Pregnancy Cohort) (Raine)
			Youth Eating Patterns study (YEP)
Belgium	1	1	
Brazil	1	1	1993 Pelotas (Brazil) birth cohort study (Pelotas)
Finland	2	1	
France	1	1	French longitudinal study of growth and nutrition (FLSGN)
Greece	1	1	
Netherlands	16	2	Amsterdam Growth and Health Longitudinal Study (AGHLS)
			Tracking Adolescents’ Individual Lives Survey (TRAILS)
Nigeria	1	1	
Norway	10	3	Norwegian Longitudinal Health Behaviour Study (NLHB)
			Oslo Youth Study (OYS)
Philippines	2	1	Cebu Longitudinal Health and Nutrition Study (CLHNS)
South Africa	2	1	Birth to Twenty study (Bt20)
Spain	1	1	
Sweden	3	3	European Youth Heart Study (EYHS)
UK	4	2	Health and Behaviour in Teenagers Study (HABITS)
			Northern Ireland Young Hearts Project (YH)
US	40	15	Coronary Artery Risk Development in Young Adults Study (CARDIA)
			Growing Up Today Study II (GUTS2)
			Identifying Determinants of Eating and Activity (IDEA) and the Etiology of Childhood Obesity (ECHO).
			Iowa Bone Development Study (IBDS)
			National Heart, Lung, and Blood Institute Growth and Health Study (NGHS)
			National Longitudinal Study of Adolescent Health (Add Health)
			National Longitudinal Survey of Youth (NLSY97)
			NEXT Generation Health Study (NEXT)
			Project Eating and Activity in Teens (Project EAT) (I, II & III)
			Study of Latino Adolescents at Risk for Diabetes (SOLAR)
Total	98	40	

Table 4 presents further details on each study, including both named and unnamed studies. All studies reported on data from after 1980, as per our inclusion criteria, but most of the studies report on data collected more recently, from the 1990s and 2000s. Notable exceptions include the Amsterdam Growth and Health Longitudinal Study (AGHLS), the Coronary Artery Risk Development in Young Adults Study (CARDIA), the Northern Ireland Young Hearts Project (YH) and the Oslo Youth Study (OYS), for which data are reported which was collected in the 1980s.

**Table 4:** Summary data from included studies

Study	Country	Sample size (n) included in the analysis (min and max within age ranges of interest)	Number of data points between 13 and 30 years (mean ages in years at each data point)	SD of age or age range at baseline or youngest age presented (years)	Year when sample were mean age 13	Method of diet data collection at each wave
Named studies						
1993 Pelotas (Brazil) birth cohort study [49]	Brazil	3915	2 (14, 18)	0·3	2007	FFQ
Adelaide Nutrition Study (ANS) [50]	Australia	106	3 (13, 15, 17)	n/r	n/r	Four day diary
Amsterdam Growth and Health Longitudinal Study (AGHLS) [51–65]	Netherlands	145 to 391	3 (16, 21, 27)	0.8	1977	Diet history interview
Birth to Twenty study (Bt20) [66, 67]	South African	1298 to 1451	3 (13, 15, 17)	0.2	2003	Questionnaire
Cebu Longitudinal Health and Nutrition Study (CLHNS) [68, 69]	Philippines	2029 to 2106	2 (15, 18)	n/r	1996	Two 24-h dietary recalls
Coronary Artery Risk Development in Young Adults Study (CARDIA) [70, 71]	US	3394 to 4278	3 (25, 26, 28)	3.6	1980	Questionnaire
European Youth Heart Study (EYHS) [72]	Sweden	179	2 (15, 21)	n/r	1996–7	Single 24-h dietary recall
French longitudinal study of growth and nutrition (FLSGN) [73]	France	94	2 (14, 16)	n/r	1998 (estimated)	Diet history interview
Growing Up Today Study II (GUTS2) [74, 75]	US	7559 to 8272	3 (13, 15, 17)	1.9	2004	FFQ
Health and Behaviour in Teenagers Study (HABITS) [76]	UK	5229 participants; numbers not reported by wave	3 (13, 14, 15)	Range 13–14	2001	Questionnaire
Identifying Determinants of Eating and Activity (IDEA) and the Etiology of Childhood Obesity (ECHO). [77]	US	562 to 666	3 (14, 16)	1.84	IDEA: 2005ECHO: 2006	Three 24-h dietary recalls
Iowa Bone Development Study (IBDS) [78]	US	255 to 428	3 (13, 15, 17, 19)	n/r	2008	Questionnaire
National Heart, Lung, and Blood Institute Growth Health Study (NGHS) [17, 79–86]	US	774 to 2371	7 (13, 14, 15, 16, 17, 18, 19)	0.6	1990–91	Three day diary
National Longitudinal Study of Adolescent Health (Add Health) [87–90]	US	597 to 16,604	3 (16, 21, 28)	1.7	1992–4	Questionnaire
National Longitudinal Survey of Youth (NLSY97) [24]	US	6244	3 (mean age n/r, range 18–22, 23–27, 27–31)	range 18–22	1993–1997	Questionnaire
NEXT Generation Health Study (NEXT) [91]	US	2172 to 2785	4 (16, 17, 18, 19)	0.03	2006–7	Questionnaire
Northern Ireland Young Hearts Project (YH) [92–94]	UK	476 to 487	3 (13, 15, 22)	1.5	1987–1991	Diet history interview
Norwegian Longitudinal Health Behaviour Study (NLHB) [95–98]	Norway	380 to 963	9 (13, 14, 15, 16, 18, 19, 21, 23, 30)	n/r	1990	Questionnaire
Oslo Youth Study (OYS) [99]	Norway	422	2 (14, 25)	range 11–17	1979	Questionnaire
Project Eating and Activity in Teens (Project EAT) [15, 100–107]	US	509 to 2134	3 (15, 20, 25)	0.8 or 1.6	1996–8	FFQ
Raine Study (Raine) [108–111]	Australia	570 to 1045	2 (14, 17)	0.2	2002–2005	FFQ
Study of Latino Adolescents at Risk for Diabetes (SOLAR) [112]	US	85	2 (14, 15)	1.6	2002 (estimated)	Two 24-h dietary recalls
Tracking Adolescents’ Individual Lives Survey (TRAILS) [113]	Netherlands	1816 to 2149	2 (13, 16)	0.6	2003	Questionnaire
Youth Eating Patterns study (YEP) [114–118]	Australia	521 to 1729	3 (13, 15, 17)	1.6	2004–5	FFQ
Unnamed studies						
Adeyanju et al. (1987) [119], Adeyanju et al. (1990) [120]	US	50 to 93	2 (14, 17)	n/r	1980	Questionnaire
Andrew et al. (2016) [121]	Australia	222 to 298	2 (14, 15)	0.9	n/r	Questionnaire
Bouziotas et al. (2003) [122]	Greece	198 to 204	2 (13, 14)	0.6	2000	Seven day diary
Bruno-Ambrosius et al. (2005) [123]	Sweden	162	3 (13, 14, 15)	range < 1 year	2000	Questionnaire
Deforche et al. (2015) [124]	Belgium	291	2 (17, 18)	0.5	2004 or 2005	FFQ
Fiorito et al. (2010) [125]	US	170	2 (13, 15)	n/r	2004	Three 24-h dietary recalls
Le et al. (2010) [126]	US	608 at baseline	4 (24, 25, 26, 27)	5.0	1990–1993	Single 24-h dietary recall
Lehtonen-Veromaa et al. (2008) [127]	Finland	142	2 (16, 20)	1.8	1997	Questionnaire
Onimawo et al. (2001) [25]	Nigeria	38	8 (mean age n/r. 18–28 at baseline, followed over 19 months)	range 18–28	n/r	Seven day diary
Ovrebo et al. (2011) [128]	Norway	583 to 606	2 (13, 15)	n/r	2002	FFQ
Pearson et al. (2011) [129]	Australia	121	2 (15, 17)	0.6	2004	Questionnaire
Racette et al. (2005) [130], Racette et al. (2008) [131]	US	204 to 208	3 (18, 19, 22)	0.3	1994–5	Questionnaire
Racette et al. (2014) [132]	US	134	2 (23, 26)	2.2	1995–97	Questionnaire
Rautava et al. (2007) [133]	Finland	142	3 (13, 16, 20)	1.8	1997	Questionnaire
Von Post-Skagegard et al. (2002) [134]	Sweden	208	3 (15, 17, 20)	0.06	1991–2	FFQ
Zarrazquin et al. (2014) [16]	Spain	285	3 (19, 20, 21)	1.4	n/r	FFQ

Each row of Table 4 represents a single study

Studies varied considerably in their sample size, ranging from 38 to 16,604 participants per wave. As can be seen in Table 4, of the larger studies with over 1000 participants (*n* = 13), many were based in the US, although other large studies include the 1993 Pelotas (Brazil) birth cohort study (Pelotas), the Birth to Twenty study (Bt20) from South Africa, the Cebu Longitudinal Health and Nutrition Study (CLHNS) from the Philippines, the Health and Behaviour in Teenagers Study (HABITS) from the UK and the Tracking Adolescents’ Individual Lives Survey (TRAILS) from the Netherlands. Four studies had fewer than 100 participants in the waves reported. For each sample, where available, we report the standard deviation or range in baseline age in Table 4. Around half of the studies (where the degree of variation in baseline age is reported) described a cohort with a narrow age range, reporting a standard deviation in age of less than one year (*n* = 15). However, it should be noted that a number of cohorts have a much wider age range, for example CARDIA, with an age range of 18–30 years at baseline and the Growing Up Today Study II (GUTS2) with an age range of 9–16 years at baseline.

### Methods of dietary assessment

**Fig. 2:**
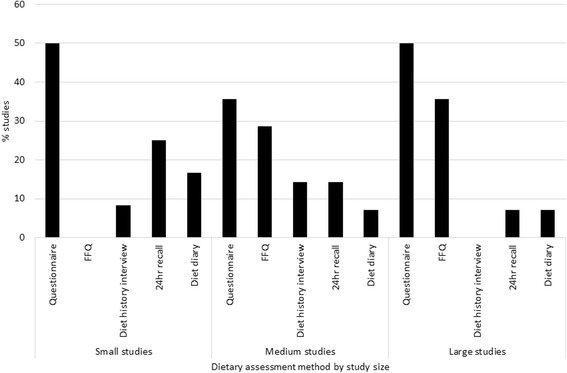
Proportion of studies using each data collection method, by study size. Note: Large studies defined as >1000 participants, medium studies: 200 < participants < 1000 and small studies: <200 participants

Studies varied in method of dietary assessment. Methods used included questionnaire items (*n* = 18), food frequency questionnaires (FFQ, *n* = 9), diet history interviews (*n* = 3), dietary recalls (*n* = 6) and diet diaries (*n* = 4). As shown in Fig. 2, large studies showed less frequent use of recall and diary methods and more frequent use of FFQs compared with medium-sized and small studies.

**Fig. 3:**
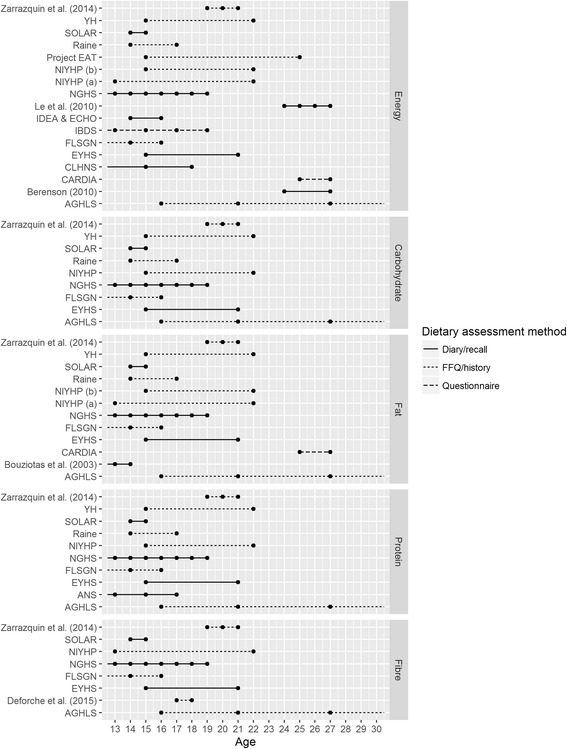
Available longitudinal data on energy and macronutrient intakes, according to mean age of the cohort, between ages 13 and 30 years. Note: Data points represent diet assessment points within each study, plotted at the mean age of study participants. Lines extend beyond data points where data (collected within our date limits) is additionally reported at mean ages below 13 or above 30. See Table 3 for full versions of all study name abbreviations

**Fig. 4:**
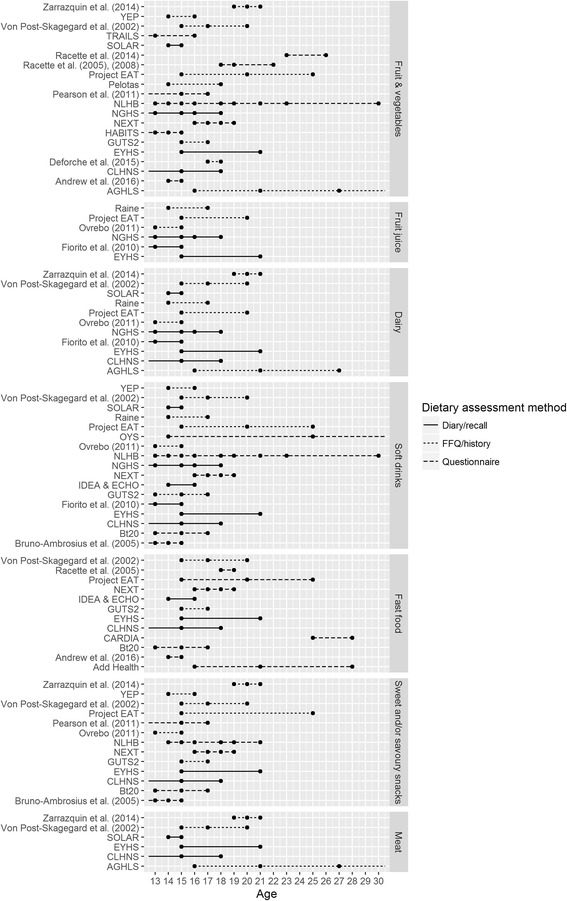
Available data on food group intakes, according to mean age of the cohort, between ages 13 and 30 years. Note: Data points represent diet assessment points within each study, plotted at the mean age of study participants. Lines extend beyond data points where data (collected within our date limits) is additionally reported at mean ages below 13 or above 30. See Table 3 for full versions of all study name abbreviations

Variation in dietary assessment method by outcome reported can also be seen from Figs. 3 and 4, below. Studies reporting on food groups, compared to macronutrient data, more frequently show dietary assessment by questionnaires rather than other methods.

### Data coverage of macronutrients and food groups

Figures 3 and 4 provide an overview of the longitudinal data available, for all macronutrients and for food groups reported on by more than five studies. Two studies could not be included in figures, due to a lack of reported information on mean age of study participants (National Longitudinal Survey of Youth (NLSY97) [24], Onimawo [25]). As noted previously, a number of studies reported on cohorts with a wide age range so that the age of study participants will vary around the mean age depicted. In the figures, studies are classified according to method of dietary assessment, presented in three groups: diaries and recalls that require participants to report all food and drink consumed on one or more specific days, FFQs and diet histories that record data on habitual dietary intake over a longer time period, or targeted questionnaires, which ask respondents to report frequency of consumption of only a limited number of food items. A small number of studies (<5 in each case) reported on consumption of additional foods that are not included in Figs. 3 and 4. These included total grains, whole grains, bread, pasta/rice, breakfast cereals, pizza/pies/pancakes, fish, spreads/oils, cakes/biscuits/puddings, nuts/legumes, or tea/coffee (see Additional file 1: Appendix 1 for a complete list of additional foods and food groups with references).

### Data coverage by age: Number and mean age of data collections

As reported in Table 4, and also visible in Figs. 3 and 4, most studies report data from only two or three time points at which the cohort is within the age range of interest. Notable exceptions include the NGHS, for which yearly repeated data on energy intake, macronutrients as well as less frequent data on a number of food groups is reported. Repeated reporting of food group intakes is also available from the NLHB and the NEXT Generation Health Study (NEXT). Where data was reported at ages outside of our age range of interest, we have indicated this by extension of the figure lines beyond the data points shown. Figures 3 and 4 also demonstrate the preponderance of available data on both macronutrients and food groups from the adolescent years and very early twenties with few studies continuing to collect relevant data into the mid and late twenties.

## Discussion

Identifying the range of available evidence to better understand changing diet through adolescence and into early adulthood is a crucial first step to inform further evidence synthesis and epidemiological analysis, and provide evidence to support appropriate targeting of dietary public health interventions. The aim of this paper was to explore how longitudinal change in diet has been conceptualised and measured between the ages of 13 and 30. To our knowledge this is the first overview of longitudinal data on food and nutrient intakes over this age range.

### Principal findings

Longitudinal dietary data were reported on total energy, the three primary macronutrients (carbohydrate, fat and protein) and fibre and a small number of food groups for cohorts between the ages of 13 and 30. While we found only seven food groups where data were reported by five or more studies, these included food groups that are of current interest to national food policies such as soft drinks, snack foods, fast food and fruits and vegetables [26, 27]. We found that the availability of data varied across the age range of interest, with considerably more studies reporting on data from the adolescent years than data from early adulthood, and very few studies continuing beyond the early twenties. Many studies provided data at only two or three time points, providing limited opportunities to gain insight into trajectories of diet over time.

### Strengths and weaknesses of this scoping review

The strength of a scoping review methodology is that it allows mapping of a heterogeneous research area and provides an overview of the data available. This is particularly useful when a body of literature has yet to be comprehensively reviewed and exhibits a large range of study designs and methodologies [20], as in this case. The review provides a comprehensive overview of published evidence on longitudinal change in diet over the period from adolescence to early adulthood across all macronutrients and food groups. However, we did not include studies that reported diet only in terms of dietary intake scores or data-driven dietary patterns, due to the challenges of making comparisons between different scores or dietary patterns.

There are, in addition, several limitations that result from the nature of a scoping review. Firstly, we do not report on the results of the diet data presented; further work will be required to analyse and synthesise the data on individual macronutrient and food group outcomes. In addition, we did not assess the quality of the evidence presented, so that, beyond information on dietary assessment method, it is not possible from this review to judge the quality of information from a given study. A further limitation derives from our literature search. Following our aim to investigate this transition period, search terms focussed on adolescence and ‘young adulthood’ or ‘early adulthood’, hence we may have missed some studies with data available in the early adult years which did not include this terminology. This limitation is difficult to overcome. However, in our hand searching we identified only one additional study, giving us a degree of confidence that our systematic search procedure was comprehensive.

### Implications of the findings

#### Populations covered by study cohorts

The context of the studies included in this review, in particular the period and country in which studies were situated, may affect the applicability of study findings to current dietary trajectories. In this review we restricted studies to those that collected data after 1980 and most of the studies included reported on data from the 1990s and 2000s, so data will be relatively applicable to current populations. However, there are known to be differences in diet and secular trends in diet between countries [28], and differences in physical and social environments across countries which may impact on diet trajectories, in particular between the US and Europe [29]. Since a large proportion of the studies in this review came from the US, and we found little data from low or middle-income countries, the generalizability of the data to other contexts may be limited.

#### Methods of dietary assessment

One of the key factors determining the quality of dietary data is the method of dietary assessment used as well as the level of compliance with data reporting among participants [30]. Studies found in this review reported use of a range of different methods which have different strengths and limitations in this age group [31–33]. The use of targeted questionnaire items is of particular concern for studying longitudinal changes in diet, since these items have often not be validated, and the lack of a comprehensive dietary assessment means that adjustment for misreporting based on energy intake or changes in overall levels of energy intake with age is not possible. Observed differences in methods of dietary assessment and data processing according to diet outcome or size of study will likely reflect differing aims and resources of the studies; many cohorts included in this review did not have a primary aim of studying longitudinal dietary data, rather, they were focussed on studying relationships between aspects of diet and anthropometric or health-related outcomes. In these large-scale cohort studies, selection of data collection instruments will be a trade-off between feasibility, acceptability, cost, validity of results and other priorities [31]. Future evidence synthesis on this topic should take account of dietary assessment method in methodological quality assessments to aid interpretation.

Future primary studies will need to consider carefully the method of diet measurement used in order to achieve valid measurement of change in dietary intake over this age range. All methods for self-reported diet data collection present limitations, frequently resulting in underreporting of total energy intake when compared to objective measures [34]. Reviews of dietary assessment methodologies used in adolescence are inconclusive as to the best method of measuring habitual intake of macronutrients and food groups among this age group [31–33]. Diet diaries have been shown to result in greater under-reporting among adolescents than adult populations, with increased underreporting with age through adolescence, and across multiple days of diary entry [32, 35]. By contrast, FFQs are characterised by low participant burden and are designed to give an indication of usual intake among participants, but show low agreement between estimated energy intake and a reference measure of total energy expenditure [33]. While no dietary assessment method is able to completely accurately assess habitual dietary intake, the use of multiple 24-h recalls may be preferred in adolescence as a compromise which achieves relatively high accuracy of data, while minimising participant burden and so increasing compliance [31, 33]. Web-based assessments, which have been shown to be comparable to interviewer-led multiple pass 24-h recalls [36], may increase compliance with and feasibility of data collection among a young and mobile population [36–38].

#### Data coverage of macronutrients and food groups

In this review, we identified that longitudinal dietary data were relatively frequently reported on total energy, the three primary macronutrients (carbohydrate, fat and protein) and fibre and a small number of food groups for cohorts between the ages of 13 and 30. This suggests that further systematic reviews focussing on the above macronutrients and food groups will be helpful to generate insights into the direction and magnitude of dietary changes over this period. A number of the food groups for which more data are available (for example, soft drinks) are of particular relevance to current health policy debates across many countries [39] and therefore of public health relevance to analyse further. While we did not explicitly assess variability in reporting of diet measures across papers in this study, it became apparent through the process of grouping papers according to food groups that, unlike with energy and macronutrients, there was considerable variation between studies in how foods were categorised and reported. This is likely to present a challenge for future systematic reviews, and further work will be required to harmonise data across studies. To aid future evidence synthesis, greater consistency in classification and reporting of food groups is recommended.

#### Data coverage by age: Number and mean age of data collections

We found considerably more studies reporting on data from the adolescent years than data from early adulthood, with very few studies continuing beyond the early twenties. Transition events, such as entry into the labour market, moving out of the parental home, formation of stable partner relationships, and entry to parenthood, are reported to be occurring at older ages than was seen previously, with many transitions continuing into the late-twenties [40]. Thus limited coverage of dietary data beyond the early twenties limits our ability to understand changes in diet across the adolescence to adulthood transition. Furthermore, many studies reported data at only two or three data points within our age range of interest, rather than reporting repeated longitudinal assessments over time. While such prospective data can provide information on determinants of dietary change which occur between the time points where diet is assessed, studies with more repeated measures will provide further insight into trajectories of behaviour over time, how these trajectories differ between groups of individuals and whether determinants of dietary change result in long-term trajectory modifications or only short-term changes which dissipate over time. To model individual trajectories, for example using growth modelling, requires a minimum of 4 time-points is recommended to allow enough parameters for model flexibility [41]. The findings of this scoping review suggest a need for further dietary data collection continuing into early adulthood, incorporating more frequently repeated dietary assessments to allow for diet trajectory analysis.

### Unanswered questions and future research

Overall our findings indicate that data reporting on longitudinal dietary change from adolescence to early adulthood are available for several macronutrients and food groups, and further systematic reviews that focus on these macronutrients or food groups will be useful to generate understanding of the direction and magnitude of dietary changes over this period. However, this review highlights significant gaps and limitations in the data available. In particular there is a paucity of longitudinal data continuing from adolescence into the mid and late twenties and few studies that report regular follow-up of participants over more than three waves. Further data collection will thus be essential to develop a thorough understanding of trajectories in dietary behaviour across this life stage. Other papers have additionally highlighted the importance of further study of adolescent nutrition [1] and the gap in available evidence on change in dietary behaviours in early adulthood [6], suggesting that researchers are now recognising the importance of this transitional life stage.

One contributing factor in the paucity of evidence is the challenge of collecting data from individuals of this age range. Particular challenges include high levels of underreporting in this age group [42], as well as difficulties of retention of adolescents and young adults in longitudinal studies. Low retention may be related to low motivation to contribute to research among this age group, or to the difficulty of tracking individuals who are frequently relocating [43, 44]. Despite these challenges, there has been recent progress in addressing such data collection challenges through technological innovations including the use of photographic methods which can calculate food intakes automatically [45] and online dietary recall which may reduce participant burden as well as lowering data collection and processing costs [43, 46, 47]. In addition, recent developments in social media have been found useful in participant recruitment [48], and may also prove useful in tracking participants longitudinally due to consistency of social media contact information over time. Together, these advances may alleviate some of the barriers to longitudinal data collection in this age group, suggesting an opportunity for new efforts to collect and analyse dietary data and study the determinants of dietary behaviour change in this age range.

## Conclusion

In this scoping review we have shown that there is limited longitudinal data available on diet between the ages of 13 and 30. While there is some data available across the macronutrients and key food groups, data availability varies significantly by country and the methods of diet data collection used may have implications for data quality and potential for future evidence synthesis. We found little repeated longitudinal data across the adolescence to adult transition, with very little data reported beyond the early twenties. Recent advances in data collection methodology and use of social media may facilitate new data collection to allow further advances in understanding of dietary change across this age range and its determinants.

## Additional file

Additional file 1: Table S1. Medline search strategy, **Appendix 1.** Additional foods reported. (DOCX 66 kb)

## Abbreviations

Add Health: National Longitudinal Study of Adolescent Health
AGHLS: Amsterdam Growth and Health Longitudinal Study
ANS: Adelaide Nutrition Study
Bt20: Birth to Twenty study
CARDIA: Coronary Artery Risk Development in Young Adults Study
CLHNS: Cebu Longitudinal Health and Nutrition Study
ECHO: Etiology of Childhood Obesity
EYHS: European Youth Heart Study
FFQ: Food frequency questionnaire
FLSGN: French longitudinal study of growth and nutrition
GUTS2: Growing Up Today Study II
HABITS: Health and Behaviour in Teenagers Study
IBDS: Iowa Bone Development Study
IDEA: Identifying Determinants of Eating and Activity
NEXT: NEXT Generation Health Study
NGHS: National Heart, Lung, and Blood Institute Growth and Health Study
NLHB: Norwegian Longitudinal Health Behaviour Study
NLSY97: National Longitudinal Survey of Youth
OYS: Oslo Youth Study
Pelotas: 1993 Pelotas (Brazil) birth cohort study
Project EAT: Project Eating and Activity in Teens
Raine: Raine Study (The Western Australian Pregnancy Cohort)
SOLAR: Study of Latino Adolescents at Risk for Diabetes
TRAILS: Tracking Adolescents’ Individual Lives Survey
WHO: World Health Organization
YEP: Youth Eating Patterns study
YH: Northern Ireland Young Hearts Project
