# Supplementary material for: Change in diet in the period from adolescence to early adulthood: a systematic scoping review of longitudinal studies
Source: Int J Behav Nutr Phys Act. 2017 May 4;14:60. doi: 10.1186/s12966-017-0518-7 (PMC5418762; doi:10.1186/s12966-017-0518-7)
Supplement: Additional file 1: Table S1. — Medline search strategy, Appendix 1. Additional foods reported. (DOCX 66 kb) [file 12966_2017_518_MOESM1_ESM.docx]

**Change in diet in the period from adolescence to early adulthood: a systematic scoping review of longitudinal studies**

Eleanor M Winpenny, Tarra L Penney, Kirsten Corder, Martin White, Esther MF van Sluijs

**Table S1: Medline search strategy**

| 1 | Diet Outcomes  Physical activity outcomes | food*[Title/Abstract] OR nutrient*[Title/Abstract] OR macronutrient*[Title/Abstract] OR “energy intake”[Title/Abstract] OR diet[Title/Abstract] OR diets[Title/Abstract] OR “dietary”[Title/Abstract] OR nutrition[Title/Abstract] OR nutritional[Title/Abstract] OR fruit[Title/Abstract] OR vegetable[Title/Abstract] OR fruits[Title/Abstract] OR vegetables[Title/Abstract] OR snack*[Title/Abstract] OR “soft drink*”[Title/Abstract] OR soda[Title/Abstract] OR SSB[Title/Abstract] OR SSBs[Title/Abstract] OR salt[Title/Abstract] OR sugar*[Title/Abstract] OR "Food"[Mesh] OR "Beverages"[Mesh] OR diet[Mesh] OR "Nutrition Surveys"[Mesh] OR "Diet Records"[Mesh] OR "Dietary Fats"[Mesh] OR "Dietary Proteins"[Mesh] OR "Dietary Carbohydrates "[Mesh] OR "Micronutrients"[Mesh]  OR “Exercise”[MeSH] OR “Sports”[Mesh] OR "physical activity"[Title/Abstract] OR "physical activities"[Title/Abstract] OR “physically active”[Title/Abstract] OR "active transport"[Title/Abstract] OR "active travel"[Title/Abstract] OR exercise*[Title/Abstract] OR cycle[Title/Abstract] OR cycling[Title/Abstract] OR walk*[Title/Abstract] OR sport*[Title/Abstract] OR "energy expenditure"[Title/Abstract] |
| --- | --- | --- |
| 2 | Longitudinal | longitudinal[Title/Abstract] OR cohort[Title/Abstract] OR prospective[Title/Abstract] OR “follow-up stud*”[Title/Abstract] OR “follow up stud*”[Title/Abstract] OR tracking[Title/Abstract] OR "Follow-Up Studies"[Mesh] OR “Prospective Studies”[Mesh] OR “Longitudinal Studies”[Mesh] |
| 3 | Age range | adolescent*[Title/Abstract] OR adolescence[Title/Abstract] OR teen*[Title/Abstract] OR student*[Title/Abstract] OR “young adult*”[Title/Abstract] OR “young adulthood”[Title/Abstract] OR “early adulthood”[Title/Abstract] OR “emerging adulthood”[Title/Abstract] OR youth*[Title/Abstract] OR “young people”[Title/Abstract] OR freshman[Title/Abstract] OR freshmen[Title/Abstract] |
| 4 | Additional filters | English[lang]  Restrict to publication year 1980 or after |
| 5 |  | 1 AND 2 AND 3 AND 4 |

**Appendix 1**

Additional foods reported with references to the papers in which they were reported:

Tea/coffee^1–4^

Bread^4–6^

Cakes, biscuits, pastries, puddings^4,6,7^

Pasta, rice, potatoes^4,6^

Pizza, pies, pancakes^5,6^

Total grains^8^

Whole grains^8–12^

Cereal^6,7,13,14^

Fish^5,7,11^

Spreads, oils^4,6,7^

Fried foods^5,15,16^

Nuts and legumes^7,11^

Grains^17^

Refined grains^9^

Cod-liver oil^5^

Vitamins and minerals^6,17–42^

**References**

1. Fiorito LM, Marini M, Mitchell DC, Smiciklas-Wright H, Birch LL. Girls’ Early Sweetened Carbonated Beverage Intake Predicts Different Patterns of Beverage and Nutrient Intake across Childhood and Adolescence. *J Am Diet Assoc*. 2010;110(4):543-550.

2. Nelson MC, Neumark-Sztainer D, Hannan PJ, Story M. Five-year longitudinal and secular shifts in adolescent beverage intake: findings from project EAT (Eating Among Teens)-II. *J Am Diet Assoc*. 2009;109(2):308-312.

3. Striegel-Moore RH, Thompson D, Affenito SG, et al. Correlates of beverage intake in adolescent girls: the National Heart, Lung, and Blood Institute Growth and Health Study. *J Pediatr*. 2006;148(2):183-187.

4. von Post-Skagegård M, Samuelson G, Karlström B, Mohsen R, Berglund L, Bratteby L-E. Changes in food habits in healthy Swedish adolescents during the transition from adolescence to adulthood. *Eur J Clin Nutr*. 2002;56(6):532-538.

5. Øvrebø EM. Food habits of school pupils in Tromso, Norway, in the transition from 13 to 15 years of age. *Int J Consum Stud*. 2011;35(5):520-528.

6. Patterson E, Wärnberg J, Kearney J, Sjöström M. The tracking of dietary intakes of children and adolescents in Sweden over six years: the European Youth Heart Study. *Int J Behav Nutr Phys Act*. 2009;6(91).

7. Zarrazquin I, Torres-Unda J, Ruiz F, et al. Longitudinal study: lifestyle and cardiovascular health in health science students. *Nutr Hosp*. 2014;30(5):1144-1151.

8. Burgess-Champoux TL, Larson NI, Neumark-Sztainer DR, Hannan PJ, Story MT. Longitudinal and secular trends in adolescent whole-grain consumption, 1999-2004. *Am J Clin Nutr*. 2010;91(1):154-159.

9. Davis JN, Alexander KE, Ventura EE, Toledo-Corral CM, Goran MI. Inverse relation between dietary fiber intake and visceral adiposity in overweight Latino youth. *Am J Clin Nutr*. 2009;90:1160-1166.

10. Lipsky LM, Haynie DL, Liu D, et al. Trajectories of eating behaviors in a nationally representative cohort of U.S. adolescents during the transition to young adulthood. *Int J Behav Nutr Phys Act*. 2015;12(138).

11. van de Laar RJJ, Stehouwer CDA, van Bussel BCT, Prins MH, Twisk JWR, Ferreira I. Adherence to a Mediterranean dietary pattern in early life is associated with lower arterial stiffness in adulthood: The Amsterdam Growth and Health Longitudinal Study. *J Intern Med*. 2013;273(1):79-93.

12. van de Laar RJJ, Stehouwer CDA, van Bussel BCT, et al. Lower lifetime dietary fiber intake is associated with carotid artery stiffness : the Amsterdam Growth and Health Longitudinal Study. *Am J Clin Nutr*. 2012;96:14-23.

13. Barton BA, Eldridge AL, Thompson D, et al. The relationship of breakfast and cereal consumption to nutrient intake and body mass index: The National Heart, Lung, and Blood Institute Growth and Health Study. *J Am Diet Assoc*. 2005;105(9):1383-1389.

14. Franko DL, Albertson AM, Thompson DR, Barton BA. Cereal consumption and indicators of cardiovascular risk in adolescent girls. *Public Health Nutr*. 2011;14(4):584-590.

15. Larson NI, Neumark-Sztainer D, Hannan PJ, Story M. Trends in Adolescent Fruit and Vegetable Consumption, 1999–2004. *Am J Prev Med*. 2007;32(2):147-150.

16. Racette SB, Deusinger SS, Strube MJ, Highstein GR, Deusinger RH. Weight changes, exercise, and dietary patterns during freshman and sophomore years of college. *J Am Coll Heal*. 2005;53(November):245-251.

17. Moore LL, Singer MR, Qureshi MM, Bradlee ML, Daniels SR. Food group intake and micronutrient adequacy in adolescent girls. *Nutrients*. 2012;4(11):1692-1708.

18. Adeyanju M. Adolescent health status, behaviors and cardiovascular disease. *Adolescence*. 1990;25(97):155-169.

19. Adeyanju M, Creswell WH. The relationship among attitudes, behaviors, and biomedical measures of adolescents “at risk” for cardiovascular disease. *J Sch Heal*. 1987;57(8):326-331.

20. Affenito SG, Thompson DR, Barton BA, et al. Breakfast consumption by African-American and white adolescent girls correlates positively with calcium and fiber intake and negatively with body mass index. *J Am Diet Assoc*. 2005;105(6):938-945.

21. Affenito SG, Thompson DR, Franko DL, et al. Longitudinal Assessment of Micronutrient Intake among African-American and White Girls: The National Heart, Lung, and Blood Institute Growth and Health Study. *J Am Diet Assoc*. 2007;107(7):1113-1123.

22. Black LJ, Allen KL, Jacoby P, et al. Low dietary intake of magnesium is associated with increased externalising behaviours in adolescents. *Public Health Nutr*. 2015;18(10):1824-1830.

23. Black LJ, Burrows SA, Jacoby P, et al. Vitamin D status and predictors of serum 25-hydroxyvitamin D concentrations in Western Australian adolescents. *Br J Nutr*. 2014;112(7):1154-1162.

24. Boon N, Koppes LLJ, Saris WHM, Van Mechelen W. The relation between calcium intake and body composition in a Dutch population: The Amsterdam Growth and Health Longitudinal Study. *Am J Epidemiol*. 2005;162(1):27-32.

25. Deforche B, Van Dyck D, Deliens T, De Bourdeaudhuij I. Changes in weight, physical activity, sedentary behaviour and dietary intake during the transition to higher education: a prospective study. *Int J Behav Nutr Phys Act*. 2015;12(1):16.

26. Deheeger M, Bellisle F, Rolland-Cachera MF. The French longitudinal study of growth and nutrition: data in adolescent males and females. *J Hum Nutr Diet*. 2002;15(6):429-438.

27. Gallagher AM, Robson PJ, Livingstone MBE, et al. Tracking of energy and nutrient intakes from adolescence to young adulthood: the experiences of the Young Hearts Project, Northern Ireland. *Public Health Nutr*. 2006;9(8):1027-1034.

28. Kemper HCG, Post GB, Welten DC, van Mechelen W, Twisk JWR. What are the effects of calcium intake on bone health in young males and females - analysis of data from the Amsterdam Growth and Health Longitudinal Study. *Acta Kinesiol Univ Tartu*. 2001;6:57-74.

29. Larson NI, Neumark-Sztainer D, Harnack L, Wall M, Story M, Eisenberg ME. Calcium and Dairy Intake: Longitudinal Trends during the Transition to Young Adulthood and Correlates of Calcium Intake. *J Nutr Educ Behav*. 2009;41(4):254-260.

30. Lehtonen-Veromaa M, Möttönen T, Leino A, Heinonen OJ, Rautava E, Viikari J. Prospective study on food fortification with vitamin D among adolescent females in Finland: minor effects. *Br J Nutr*. 2008;100:418-423.

31. Magarey AM, Boulton TJC, Chatterton BE, Schultz C, Nordin BEC. Familial and environmental influences on bone growth from 11-17 years. *Acta Paediatr*. 1999;88(11):1204-1210.

32. Onimawo IA. Inter-individual variations in energy and nutrient intake among young Nigerian adults. *Ecol Food Nutr*. 2001;40(2):127-141.

33. O’Sullivan TA, Bremner AP, Mori TA, et al. Regular fat and reduced fat dairy products show similar associations with markers of adolescent cardiometabolic health. *Nutrients*. 2016;8:22.

34. Parker CE, Vivian WJ, Oddy WH, Beilin LJ, Mori TA, O’Sullivan TA. Changes in dairy food and nutrient intakes in Australian adolescents. *Nutrients*. 2012;4:1794-1811.

35. Post B, Kemper HC, Storm-Van Essen L. Longitudinal changes in nutritional habits of teenagers: differences in intake between schooldays and weekend days. *Br J Nutr*. 1987;57:161-176.

36. Post GB, Kemper HCG. Nutrient intake and biological maturation during adolescence. The Amsterdam growth and health longitudinal study. *Eur J Clin Nutr*. 1993;47:400-408.

37. Post GB, de Vente W, Kemper HCG, Twisk JWR. Longitudinal trends in and tracking of energy and nutrient intake over 20 years in a Dutch cohort of men and women between 13 and 33 years of age: The Amsterdam growth and health longitudinal study. *Br J Nutr*. 2001;85:375-385.

38. Rautava E, Lehtonen-Veromaa M, Kautiainen H, et al. The reduction of physical activity reflects on the bone mass among young females: A follow-up study of 142 adolescent girls. *Osteoporos Int*. 2007;18(7):915-922.

39. Twisk JWR, Staal BJ, Brinkman MN, Kemper HCG, van Mechelen W. Tracking of lung function parameters and the longitudinal relationship with lifestyle. *Eur Respir J*. 1998;12(3):627-634.

40. Welten DC, Kemper HCG, Post GB, Van Staveren WA, Twisk JWR. Longitudinal development and tracking of calcium and dairy intake from teenager to adult. *Eur J Clin Nutr*. 1997;51(9):612-618.

41. Welten DC, Kemper HCG, Post GB, et al. Weight-Bearing Activity During Youth Is a More Important Factor for Peak Bone Mass than Calcium Intake. *J Bone Miner Res*. 1994;9(7):1089-1096.

42. White J, Jago R, Thompson JL. Dietary risk factors for the development of insulin resistance in adolescent girls: a 3-year prospective study. *Public Health Nutr*. 2014;17(2):361-368.
